# *Staphylococcus aureus* Extracellular Vesicles Elicit an Immunostimulatory Response *in vivo* on the Murine Mammary Gland

**DOI:** 10.3389/fcimb.2018.00277

**Published:** 2018-08-22

**Authors:** Natayme R. Tartaglia, Koen Breyne, Evelyne Meyer, Chantal Cauty, Julien Jardin, Denis Chrétien, Aurélien Dupont, Kristel Demeyere, Nadia Berkova, Vasco Azevedo, Eric Guédon, Yves Le Loir

**Affiliations:** ^1^STLO, INRA, Agrocampus Ouest, Rennes, France; ^2^Federal University of Minas Gerais, Belo Horizonte, Brazil; ^3^Department of Pharmacology, Toxicology and Biochemistry, Faculty of Veterinary Medicine, Ghent University, Merelbeke, Belgium; ^4^CNRS, Institut de Génétique et Développement de Rennes - UMR 6290, Université de Rennes, Rennes, France; ^5^CNRS, INSERM, Biologie, Santé, Innovation Technologique de Rennes - UMS 3480, Université de Rennes, Rennes, France

**Keywords:** mastitis, *Staphylococcus aureus*, EV, membrane vesicle, immunomodulation, pathogenesis, intramammary infection, virulence factor

## Abstract

*Staphylococcus aureus* is a major pathogen responsible for bovine mastitis, the most common and costly disease affecting dairy cattle. *S. aureus* naturally releases extracellular vesicles (EVs) during its growth. EVs play an important role in the bacteria-bacteria and bacteria-host interactions and are notably considered as nanocarriers that deliver virulence factors to the host tissues. Whether EVs play a role in a mastitis context is still unknown. In this work, we showed that *S. aureus* Newbould 305 (N305), a bovine mastitis isolate, has the ability to generate EVs *in vitro* with a designated protein content. Purified *S. aureus* N305-secreted EVs were not cytotoxic when tested *in vitro* on MAC-T and PS, two bovine mammary epithelial cell lines. However, they induced the gene expression of inflammatory cytokines at levels similar to those induced by live *S. aureus* N305. The *in vivo* immune response to purified *S. aureus* N305-secreted EVs was tested in a mouse model for bovine mastitis and their immunogenic effect was compared to that of live *S. aureus* N305, heat-killed *S. aureus* N305 and to *S. aureus* lipoteichoic acid (LTA). Clinical and histopathological signs were evaluated and pro-inflammatory and chemotactic cytokine levels were measured in the mammary gland 24 h post-inoculation. Live *S. aureus* induced a significantly stronger inflammatory response than that of any other condition tested. Nevertheless, *S. aureus* N305-secreted EVs induced a dose-dependent neutrophil recruitment and the production of a selected set of pro-inflammatory mediators as well as chemokines. This immune response elicited by intramammary *S. aureus* N305-secreted EVs was comparable to that of heat-killed *S. aureus* N305 and, partly, by LTA. These results demonstrated that *S. aureus* N305-secreted EVs induce a mild inflammatory response distinct from the live pathogen after intramammary injection. Overall, our combined *in vitro* and *in vivo* data suggest that EVs are worth to be investigated to better understand the *S. aureus* pathogenesis and are relevant tools to develop strategies against bovine *S. aureus* mastitis.

## Introduction

Mastitis is an inflammatory response of the mammary gland that often results from a bacterial infection and that induces local to systemic symptoms in small and large ruminants (Bradley, 2002; Le Maréchal et al., 2011b). In dairy farms, mastitis severely impacts both the animal health and the quality of milk, causing important economic losses in the dairy industry (Le Maréchal et al., 2011b; Peton and Le Loir, 2014). The Gram-positive pathogen *Staphylococcus aureus* is one of the most important etiological agent of mastitis worldwide (Bradley, 2002; Le Maréchal et al., 2011b). Signs of *S. aureus* mastitis range from subclinical to gangrenous infection in ruminants and rely on strain-specific features such as the production and secretion of specific virulence factors that increase invasiveness or enable the mammary epithelial colonization of *S. aureus* (Le Maréchal et al., 2011a,c; Peton and Le Loir, 2014). Clearance of *S. aureus* from the infected udder is impaired as the pathogen is able to adhere, internalize, survive and multiply into the mammary epithelium (Alekseeva et al., 2013; Bouchard et al., 2013; Peton et al., 2014). This ability of *S. aureus* to induce chronic infections negatively affects animals and notably, *S. aureus* mastitis are reportedly difficult to cure and show a high recurrence rate (Conlon, 2014; Peton and Le Loir, 2014; Peton et al., 2014).

The bovine strain *S. aureus* Newbould 305 (Prasad and Newbould, 1968; Bouchard et al., 2012), hereafter referred to as *S. aureus* N305, has been used as a model strain for *S. aureus* mastitis in numerous studies including several from our group (Bouchard et al., 2013; Breyne et al., 2014, 2017a,b; Peton et al., 2016). Despite many efforts dedicated to understand the pathogenesis of *S. aureus* mastitis, the infectious process is still poorly understood and a better knowledge on host-pathogen interactions is required to allow the development of effective preventive or curative strategies. *S. aureus* secretes many virulence factors as well as exports both envelope-associated proteins through classical Sec-dependent pathways and cytoplasmic proteins through non-classical secretion mechanisms (Bendtsen et al., 2005; Hecker et al., 2010). One of these latter mechanisms is the release of extracellular vesicles (EVs) which has been extensively described in eukaryotes (van der Pol et al., 2012; Roy et al., 2018). These EVs are spherical nano-sized particles with a lipid bilayer secreted naturally by pathogenic and non-pathogenic bacteria from budding of the cellular membranes (Prados-Rosales et al., 2011; Deatherage and Cookson, 2012; Al-Nedawi et al., 2015). Donor cells use EVs to transport various proteins which can be delivered to local or distant cellular targets to interact with and modify them. The first evidence of this novel secretion process was obtained in Gram-negative bacteria already in the 1960s (Knox et al., 1966; Work et al., 1966; Chatterjee and Das, 1967). EVs are now recognized as important vehicles of intra- and inter-species cellular communication across all three kingdoms of life (Deatherage and Cookson, 2012; Celluzzi and Masotti, 2016). The protein content of bacterial vesicles includes factors involved in virulence, biofilm formation, modulation of the host immune response, resistance to antibiotics, bacterial survival and intra- and interspecies communication and cooperation (MacDonald and Kuehn, 2012; Brown et al., 2015; Kim et al., 2015). Most studies have been conducted on Gram-negative bacteria (Horstman and Kuehn, 2000; Ellis and Kuehn, 2010; José Fábrega et al., 2016). Consequently, our knowledge regarding Gram-positive EVs still remains limited (Brown et al., 2015). Since 2009, a few works reported the production and secretion of EVs by *S. aureus*, with particular emphasis on their protein content characterization and their impact on host cells (Lee et al., 2009, 2013b; Gurung et al., 2011; Hong et al., 2011; Kim et al., 2012; Thay et al., 2013; Choi et al., 2015; Jeon et al., 2016; Bae et al., 2017; He et al., 2017; Im et al., 2017; Jun et al., 2017; Askarian et al., 2018). In analogy with Gram-negative bacteria *S. aureus* EVs harbor, *inter alia*, numerous virulence factors, can have cytotoxic effects on host cells *in vitro* (Gurung et al., 2011; Jeon et al., 2016) and trigger a pro-inflammatory response both *in vitro* and *in vivo* (Hong et al., 2011; Kim et al., 2012).

However, the potential contribution of *S. aureus* EVs to bacterial pathogenesis has only been explored for human isolates. Therefore, in the present report we aimed to at first characterize EVs produced by the bovine mastitis strain *S. aureus* N305 to investigate their role in the context of mastitis. To obtain this aim, we evaluated whether these purified EVs are capable to induce a stimulation of the host immune response comparable to either live or heat-killed *S. aureus* N305 and LTA. Our data demonstrate that *S. aureus* N305-secreted EVs induce an innate immune response on bovine mammary epithelial cells *in vitro* and a mild pro-inflammatory response in mammary tissues *in vivo*.

## Materials and methods

### Bacterial strain and growth conditions

*S. aureus* N305 (ATCC 29740) was grown in Brain Heart Infusion (BHI) (Difco, pH 7.4) broth at 37°C under vigorous shaking (150 rpm/min). The phases of bacterial growth were determined by measurement of optical density at 600 nm (OD_600_) and routinely the colony forming units (CFU) were counted on BHI agar using the micromethod (Baron et al., 2006).

### Mammary epithelial cell lines and culture conditions

The bovine mammary epithelial cell line MAC-T (Nexia Biotechnologies, Quebec, Canada) was cultured in Dulbecco's modified eagle medium (DMEM) (D. Dutscher) supplemented with 10% heat-inactivated fetal calf serum, 40 U/mL penicillin, 40 μg/mL streptomycin (LONZA), and 5 μg/mL insulin (Sigma-Aldrich). The bovine mammary epithelial cell line PS (INRA, Tours, France) (Roussel et al., 2015) was cultured in mammary epithelial cells growth medium (GM) which contain Advanced DMEM/F12 (Gibco) supplemented with 20 mM HEPES buffer (Fisher Scientific), 2 mM L-glutamine (Gibco), 1 μg/mL hydrocortisone (Sigma-Aldrich), 10 ng/mL insulin-like growth factor 1 (Preprotech), 5 ng/mL fibroblast growth factor (Preprotech), 5 ng/mL epidermal growth factor (Sigma-Aldrich) (Roussel et al., 2015). Infections of PS cells were performed with stimulation medium (SM) without growth factors (Roussel et al., 2015). MAC-T and PS cells were incubated at 37°C in humidified incubator with 5% CO_2_. They were cultured to a confluent monolayer (80%), treated with 0.05% trypsin (PAN-Biotech) and suspended in fresh medium.

### Purification of *S. aureus* N305-secreted EVs from culture supernatants

EVs were purified from *S. aureus* N305 culture supernatants using a method adapted from (Gurung et al., 2011). Sub-cultured cells at the end of exponential phase were diluted 1:1,000 in 1 L of fresh BHI medium and were grown until the stationary phase. After the cells were pelleted at 6,000 *g* for 15 min, the supernatant fraction was filtered through a 0.22 μm vacuum filter (PES) and the filtrate was concentrated around 100-fold using Amicon ultrafiltration system (Millipore) with 100 kDa filter. The resulting filtrate was subjected to ultracentrifugation at 150,000 *g* for 120 min at 4°C and were applied to a discontinuous sucrose density gradient (8–68%). After centrifugation at 100,000 *g* for 150 min at 4°C, each fraction of the gradient was collected. The fractions with density around 1.08–1.13 g/cm^3^ were then recovered by sedimentation at 150,000 *g* for 120 min and suspended in Tris-Buffered Saline (TBS) (150 mM NaCl; 50 mM Tris-Cl, pH 7.5). Purified EVs were checked for absence of bacterial contamination and stored at −20°C before use. The EVs amount were measured based on protein concentration using the Bradford reagent (Bio-Rad) and visualized by SDS-PAGE. Hereafter, the *S. aureus*-secreted vesicle dose correspond to the quantity of *S. aureus*-secreted vesicle proteins.

### Negative staining electron microscopy (EM)

Negative staining electron microscopy was performed at the Microscopy Rennes Imaging Center platform (MRic TEM) (University of Rennes 1, Rennes, France). Purified EVs were applied to copper grids and were negatively stained with 2% uranyl acetate as previously described (Gurung et al., 2011). The samples were visualized on a transmission electron microscope Jeol 1400 TEM (Jeol, Tokyo, Japan) operating at 120 kv accelerating voltage.

### Cryo-electron tomography (Cryo-ET)

Vitrification of purified EVs was performed using an automatic plunge freezer (EM GP, Leica) under controlled humidity and temperature (Dubochet and McDowall, 1981). Mix-capped gold nanoparticles of 10 nm in diameter (Duchesne et al., 2008) were added to the sample at a final concentration of 80 nM to be used as fiducial markers. The samples were deposited to glow-discharged electron microscope grids followed by blotting and vitrification by rapid freezing into liquid ethane. Grids were transferred to a single-axis cryo-holder (model 626, Gatan) and were observed using a 200 kV electron microscope (Tecnai G^2^ T20 Sphera, FEI) equipped with a 4k × 4k CCD camera (model USC 4000, Gatan). Single-axis tilt series, typically in the angular range ±60°, were acquired under low electron doses (~0.3 e^−^/Å^2^) using the camera in binning mode 2 and at a nominal magnifications of 29,000x. Tomograms were reconstructed using the graphical user interface eTomo from the IMOD software package (Mastronarde, 1997). Slices through the tomograms were extracted using the graphical user interface 3dmod of the IMOD package. Measurements were performed using the measuring tools available in the slicer panel of 3dmod.

### Size distribution of *S. aureus* N305-secreted EVs

The size distribution of EVs was estimated by three different methods: nanoparticle tracking analysis (NTA), tunable resistive pulse sensing (TRPS) and cryo-EM. NTA analysis was carried out using a NanoSight NS300 (Malvern Instruments, United Kingdom). EVs were thawed and diluted in TBS at 1:10,000 until an optimum visualization of a maximum number of vesicles. Data was analyzed by NTA 3.0 software (Malvern Instruments). All measurements were performed at 22°C. TRPS analysis was carried out using the IZON qNano system (Izon Science). EVs were diluted 1:100 and applied to qNano instrument (Izon Science) at 22°C using a nanopore NP140 after the calibration of the system with 70 nm standard carboxylated polystyrene particles (CPC70). Finally, the diameter of vesicles was measured using the images obtained by Cryo-EM from 90 round vesicles using the measuring tools available in the slicer panel of 3dmod (IMOD package).

### In-solution digestion and identification of proteins in *S. aureus* N305-secreted EVs

Three independent biological replicates of EVs, purified as described above, were digested for NanoLC-ESI-MS/MS analysis. Purified EVs (approximately 50 μg) were pelleted at 150,000 *g* for 2 h at 4°C and suspended with the solution of 6 M Guanidine-HCl (Sigma-Aldrich), 50 mM Tris-HCl (pH 8.0) (VWR C) and 2 mM DTT (Sigma-Aldrich). EVs were heated at 95°C for 20 min and cooled in 50 mM NH_4_HCO_3_ (pH 7.8) (Sigma-Aldrich). Then, samples were digested in solution using sequencing grade-modified trypsin (Promega) with the ratio 1:50 of enzyme:protein for 15 h at 37°C, as previously described by Lee et al. (2009). After digestion, the peptides were stored at −20°C until further analysis. Nano-LC experiments were performed as previously reported (Le Maréchal et al., 2011a), with minor modifications. Briefly, the peptide mixture was loaded using a Dionex U3000-RSLC nanoLC system fitted to a Q-Exactive mass spectrometer (Thermo Scientific, USA) equipped with a nano-electrospray ion source (ESI) (Proxeon Biosystems A/S). Samples were first concentrated on a PepMap 100 reverse-phase column (C18, 5 μm, 300 μm inner diameter (i.d.) by 5 mm length) (Dionex). Peptides were then separated on a reverse phase PepMap column (C18, 3 μm, 75 μm i.d. by 250 mm length) (Dionex) using solvent A [2% (v/v) acetonitrile, 0.08% (v/v) formic acid, and 0.01% (v/v) TFA in deionized water] and solvent B [95% (v/v) acetonitrile, 0.08% (v/v) formic acid, and 0.01% (v/v) TFA in deionized water]. A linear gradient from 5 to 85% of solvent B was applied for the elution at a flow rate of 0.3 μL/min. MS data was acquired in positive mode and the spectra were collected in the selected mass range 250 to 2,000 m/z at a resolution of 70,000 for MS and at a resolution of 17,500 for MS/MS spectra. The peptides were identified from the MS/MS spectra using the X! Tandem pipeline software (Langella et al., 2017), matched against the genome sequence of the *S. aureus* N305 and *S. aureus* RF122, a bovine strain associated with severe symptoms in the host (Herron-Olson et al., 2007). A minimum of two peptides per protein was imposed with a false discovery rate (FDR) of < 0.1% at the peptide level.

### Bioinformatics

The biological functions and distribution of *S. aureus* N305 EVs proteins were categorized according to the Clusters of Orthologous Groups of proteins (COGs) (Tatusov et al., 2000). The proteins identified in this study were searched against the UniProt (http://www.uniprot.org/) and NCBI (https://www.ncbi.nlm.nih.gov/) databases. Their subcellular locations were analyzed using PsortB (http://www.psort.org/psortb/) and the cleavage of the signal peptide was inferred through SignalP version 4.1 (http://www.cbs.dtu.dk/services/SignalP/) (Nielsen, 2017). The prediction of lipoproteins was performed using LipoP version 1.0 (http://www.cbs.dtu.dk/services/LipoP/) (Rahman et al., 2008) and TMHMM version 2.0 (http://www.cbs.dtu.dk/services/TMHMM/) was used to inferred the transmembrane helices in proteins. The moonlight proteins were identified using MoonProt database (Mani et al., 2015).

### Protein extraction and SDS-page electrophoresis

Proteins samples for total bacterial lysates and supernatants were extracted as previously described (Le Maréchal et al., 2009). For the extraction of proteins from the supernatant (SP), bacterial cultures were centrifuged at 7,000 *g* for 20 min at 4°C and the supernatants were filtered through a 0.45 μm filter. Then, the proteins were precipitated with 10% TCA at 4°C for 16 h and were centrifuged at 7,000 *g* for 90 min at 4°C. Protein pellets were washed with ethanol 96% and the samples were stored at −20°C. For total protein extracts (WC), cells were lysed with 200 μg/mL lysostaphin (Invitrogen) for 1 h at 37°C in Tris-EDTA buffer (Sigma-Aldrich). 10 μg of each extract (WC, SP and intact EVs) were treated for 10 min at 100°C in Laemmli buffer and separated by 12% SDS-PAGE (Laemmli, 1970) and the gel was subsequently stained with Bio-Safe Coomassie (Bio-rad).

### Eukaryotic cell viability assay

The viability of eukaryotic cells was evaluated as previously described with slight modifications (Peton et al., 2014). Briefly, MAC-T and PS cell lines were seeded in 96-well plates at densities of 10^4^ cells per well, cultured to 80% confluence and incubated for 24 h with DMEM alone (mock control) and DMEM containing triton X-100 (0.01%) (positive control) or various quantities of *S. aureus* N305 EVs (0.01, 0.1, 1, and 10 μg per well). The cell viability was evaluated using 0.5 mg/mL Thiazolyl Blue Tetrazolium Bromide (MTT) (Sigma-Aldrich) according to manufacturer's protocol. The absorbance was evaluated at 570 nm and viability was expressed using 100% viability as mock control condition.

### qRT-PCR gene expression

Confluent monolayers of PS cells were seeded in a 12-well cell culture plate at densities of 2.0 × 10^5^ cells per well. Briefly, cells were washed twice with Hank's Balanced Salt Solution (HBSS) (D. Dutscher) and incubated for 3 h with DMEM (mock control), and DMEM containing viable (N305) and heat-killed *S. aureus* N305 (N305_HK_) cells at a multiplicity of infection (MOI) of 100 bacteria per cell, 10 μg of purified staphylococcal lipoteichoic acid (LTA) (InvivoGen, USA), and 10 and 20 μg of N305 EVs. Note that 20 μg of *S. aureus* N305 EVs corresponded to the relation of 1 μg per 10^4^ cells in the viability assays. Once heat-treated for 30 min at 80°C, the samples were re-plating to ensure that all bacteria had been inactivated. After the incubation period, total RNA was extracted using RNeasy mini-kit (Qiagen) and treated with DNAse-free DNA Removal Kit (ThermoFisher Scientific) to remove residual genomic DNA according to manufacturer's instructions. RT-qPCR was carried out using first-strand cDNA synthetized from 500 ng of total RNA samples by qScript cDNA Synthesis kit (Quantabio). The PPIA (peptidyl-prolyl *cis-trans* isomerase A), RPL19 (ribosomal protein 19) and YWHA (14-3-3 phospho-serine/phospho-threonine binding protein) housekeeping genes were used as reference genes for normalization. Amplification was performed on a CFX96 real-time system (Bio-Rad, France) and the primers used in this study are listed in Table S1. The samples setups included biological triplicates and experimental triplicates. Genes considered significantly differentially expressed corresponded to those with a *P*-value < 0.05 (student's *t*-test) when compared to the mock control.

### Intraductal inoculation of *S. aureus* N305-secreted EVs in the mouse mammary gland

The *in vivo* experimental mastitis study was conducted with a comparable protocol as previously described (Brouillette et al., 2004; Le Maréchal et al., 2011a; Peton et al., 2016). It was performed in accordance with the International Guiding Principles for Biomedical Research Involving Animals under approval the Ethical Committee of the Faculty of Veterinary Medicine in the University of Ghent, Belgium (no. EC2015_127). Forty Hsd:ICR (CD-1) outbred lactating female mice (Envigo, The Netherlands) were mated with male mice and were used 12 days after birth of the offspring. The pups were separated 2 h before of the intraductal inoculation in the fourth mammary gland pair. A mixture of oxygen and isoflurane (2–3%) was used for inhalational anesthesia of the mice and a bolus of PBS-diluted Vetergesic (i.e., buprenorphine 10 μg/kg, Val d'Hony Verdifarm NV, Belgium) was administered intraperitoneally (i.p.) as analgesic prior to any surgical intervention. The mammary gland duct was exposed through a small cut at the teat tip and each sample was slowly intraductally injected at a volume of 100 μL with a 32 gauge blunted needle. Six groups of mice were simultaneously inoculated: two groups each received *S. aureus* N305-secreted EVs (at concentrations of 1 and 10 μg in phosphate-buffered saline or PBS, both *n* = 7) and compared to a negative control group (sham) receiving PBS only (*n* = 7). Three independent positive control groups were included in the study set-up for comparative purposes, a first one receiving 117 CFU of viable *S. aureus* N305 in PBS (N305, *n* = 7), a second one receiving 100 CFU of heat-killed *S. aureus* N305 in PBS (N305_HK_, *n* = 6), and a third one receiving 10 μg of lipoteichoic acid (LTA) in PBS (InvivoGen, USA) (*n* = 6). Twenty-four hours post-infection (p.i.), mice were sedated by an intraperitoneal administered mixture of ketamine (100 mg/kg Anesketin, Eurovet Animal Health BV, Bladel, The Netherlands) and xylazine (10 mg/kg; Xylazini Hydrochloridum, Val d'Hony-Verdifarm, Belgium) and, subsequently, euthanized through cervical dislocation.

### Bacterial load, cytokine profiling, and histology

Upon necropsy, all mammary glands were isolated and mechanically homogenized. A serial dilution derived from 20 μL of homogenate was plated on Tryptic Soy Agar to obtain a number of CFU per amount of tissue (g). To another 100 μL-aliquot of the homogenate 400 μL of lysis buffer supplied with protease inhibitors (200 mM NaCl, 10 mM Tris-HCl pH 7.4, 5 mM EDTA, 1% Nonidet P-40, 10% glycerol, 1 mM oxidized L-glutathion, 100 μM PMSF, 2.1 μM leupeptin, and 0.15 μM aprotinin) was added for later extraction of proteins. Mammary gland lysates were frozen overnight and centrifuged the following day at 12,250 *g* for 1 h. After recovering of the supernatant, the sample was quantified through Bio-Rad protein staining followed by spectrophotometry at 595 nm (Genesys 10S). All the samples were then adjusted to reach the same protein concentration (5 μg/μL). Selected cytokine profiling was done using a bead-based multiplex immunoassay (ProcartaPlex, Thermo Fisher Scientific) for the simultaneous quantification of IL-1α, IL-1β, IL-6, TNF-α, MCP-1, CXCL2 (MIP-2), RANTES and BAFF and specific simplex immunoassays (ProcartaPlex) for mouse CXCL1 (KC) and IL-17A. All assays were performed in accordance with the manufacturer's instructions after in house validation for mammary gland matrix. Isolated mammary glands (*n* = 2 per condition) were fixed in 3.5% buffered formaldehyde, embedded in paraffin and sections were deparaffinized and stained with hematoxylin and eosin (H&E). Mammary gland tissues were visualized at x200 and x400 magnification.

### Statistical analysis

The data were presented as mean concentration ± standard error. The differences between the animal groups were assessed using one-way analysis of variance, followed by Tukey's range test. The statistical program Prism 6 (GraphPad) was used considering significant a *P*-value lower than 0.05.

## Results

### The bovine mastitis-associated *S. aureus* strain N305 produces EVs *in vitro*

**Figure 1:**
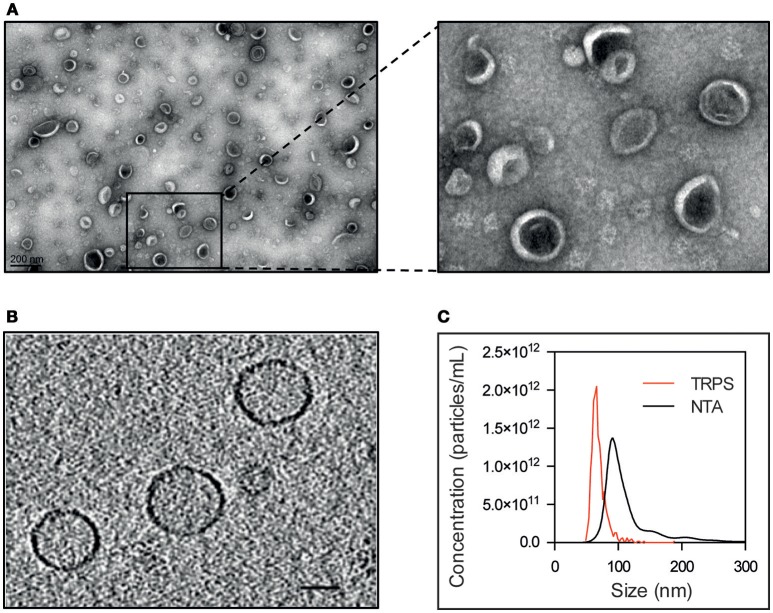
Bovine *S. aureus* Newbould 305 (N305) releases EVs *in vitro*. TEM of *S. aureus* Newbould 305 (N305) purified EVs after negative staining **(A)** and selected EVs. **(B)** Slice through a cryo-electron tomogram obtained from *S. aureus* N305 EVs. **(C)** Representative graph of size distribution of *S. aureus* N305-secreted EVs measured with tunable resistive pulse sensing (TRPS) and nanoparticle tracking analysis (NTA).

EVs secreted by *S. aureus* N305 were isolated from the cell-free supernatants of stationary phase cultures. For that purpose, we used centrifugation, filtration and density gradient ultracentrifugation, the standard method for the isolation and purification of membrane vesicles with higher purity (Yamada et al., 2012; Dauros Singorenko et al., 2017). Homogeneity and integrity of vesicles were evaluated by both negative staining electron microscopy and cryo-electron tomography (cryo-ET). Electron micrographs of purified EVs revealed nano-sized vesicular structures with a typical cup-shape (Raposo and Stoorvogel, 2013; Figure 1A). Cryo-ET analysis showed homogeneously shaped spherical particles (Figure 1B). The size distribution of EVs was estimated by three different methods: nanoparticle tracking analysis (NTA), tunable resistive pulse sensing (TRPS) and cryo-ET. Average EV sizes were 67 ± 13 nm (mean and standard deviation) for TRPS, 91 ± 23 nm for cryo-ET and 126 ± 2 nm for NTA (Figure 1C). Although average size of EVs may vary according to the analytical method due to the limitations of each methodology (van der Pol et al., 2010, 2014; Maas et al., 2015; Sitar et al., 2015), these complementary approaches highlighted the monodisperse size distribution of *S. aureus* N305 EVs. In addition, the total particle count evaluated by TRPS and NTA was similar and close to 4 × 10^9^ particles per mL of bacterial culture supernatant. These results demonstrate that the bovine mastitis-associated *S. aureus* strain N305 releases a high number of EVs homogenous in size and shape under laboratory culture conditions.

### Bovine *S. aureus* N305-secreted EVs carry virulence factors

**Figure 2:**
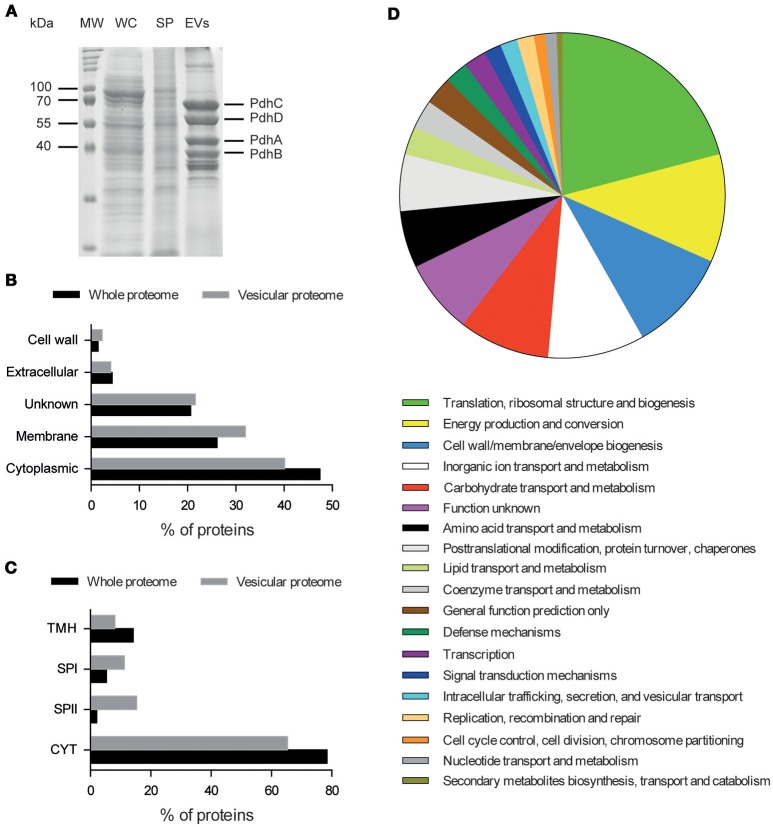
Identification and distribution of proteins associated with *S. aureus* N305-secreted EVs. **(A)** SDS-PAGE (12%) protein separation. Lanes: MW, Molecular weight standards are indicated on the left (kDa); WC, whole-cell lysates; SP, supernatant; EVs, *S. aureus* N305 EVs. Proteins from the major Blue Coomassie stained bands that correspond to PdhA, PdhB, PdhC, and PdhD, components of the pyruvate dehydrogenase complex, were identified by LC-MS/MS. **(B)** Vesicular proteome distribution compared to whole bacterial proteome based on their localization with the predictor PsortB. **(C)** Vesicular proteome distribution compared to whole bacterial proteome based on their localization with the predictor LipoP. TMH, N-terminal transmembrane helices; SPI and II, signal peptidase I or II; CYT, cytoplasmic proteins. **(D)** Protein distribution based on their COG annotation (IMG source).

In addition to their structural characterization, cargo proteins of *S. aureus* N305-secreted EVs were determined through LC-MS/MS analysis of their proteomic profiles on three independent purified samples. The pattern of proteins associated with purified *S. aureus* N305-secreted EVs differed from that of the other bacterial cell fractions (Figure 2A). A total of 222 proteins were consistently identified (Table S2), with the majority (*n* = 160) predicted to be either cytoplasmic (*n* = 89) or putatively membrane-associated (*n* = 71) (Figure 2B), showing that shedding of EVs appears to be also a pathway for protein secretion in *S. aureus* N305. The latter were overrepresented in EVs when compared to the predicted whole membrane proteome (32 vs. 26%). More than half (34/58) of the number of predicted lipoproteins from the whole proteome (i.e., proteins with a signal peptidase II cleavage site) were identified, indicative for their relative enrichment in EVs (Figure 2C).

These identified proteins were involved in various bacterial processes (Figure 2D). In comparison with the whole *S. aureus* N305 proteome, some COGs were overrepresented in *S. aureus* N305-secreted EVs related to translation, ribosomal structure and biogenesis (19.0 vs. 9.0%), energy production and conversion (10.4 vs. 5%), cell wall (9.4 vs. 5.7%), membrane and envelope biogenesis (9.4 vs. 5.7%) and defense mechanisms (5.8 vs. 2.8%). Furthermore, proteins with moonlighting abilities, such as autolysin, enolase, GAPDH and elongation factor Tu were identified. Most importantly, EVs contained numerous virulence factors (Table 1) including the immunoglobulin G-binding protein (Sbi) (Burman et al., 2008; Lee et al., 2009; Jeon et al., 2016), penicillin-binding protein (PBPs) (Lowy, 2003; Lee et al., 2009; Jeon et al., 2016), elastin binding protein (EbpS) (Park et al., 1996), the autolysin (Atl) (Lee et al., 2009; Hirschhausen et al., 2010; Jeon et al., 2016), the phenol soluble modulins (PSMs) (Cheung et al., 2014; Jeon et al., 2016).

**Table 1:** Potentially associated virulence factors identified in *S. aureus* Newbould 305-secreted EVs.

**Gene ID**	**Description**	**Function**	**References**
**ADHESION AND TISSUE DAMAGE**			
**Adhesion and internalization**			
Newbould305_1791	Fibrinogen-binding protein (FnBP)	Binds to host fibrinogen	Rivera et al., 2007
Newbould305_2258	Elastin binding protein (EbpS)	Promotes binding of soluble elastin peptides and tropoelastin to *S. aureus* cells	Park et al., 1996
**Evasion of host immune system**			
Newbould305_2589	Immunoglobulin G-binding protein (Sbi)	Interacting selectively and non-covalently with an immunoglobulin	Burman et al., 2008
**Toxins**			
Newbould305_2342	Delta-hemolysin (Hld)	Lyses erythrocytes and many other mammalian cells	Vandenesch et al., 2012
PSMA1_STAAB	Alpha-class phenol-soluble modulin (PSMα1)	Pathogenesis	
PSMA2_STAAB	Alpha-class phenol-soluble modulin alpha 2 (PSMα2)	Pathogenesis	
PSMA4_STAAB	Alpha-class phenol-soluble modulin alpha 4 (PSMα4)	Pathogenesis	
Newbould305_1816	Beta-class phenol-soluble modulin (PSMβ1)	Pathogenesis	
Newbould305_1817	Beta-class phenol-soluble modulin (PSMβ2)	Pathogenesis	
Newbould305_2380	Uncharacterized leukocidin-like protein 2	Cytolysis in other organism; Pathogenesis	
**Regulatory system**			
Newbould305_2136	Peptide methionine sulfoxide reductase regulator MsrR	Involved in SarA attenuation. Role in resistance to oxacillin and teicoplanin, as well as the synthesis of virulence factors	Rossi et al., 2003
**Poorly characterized**			
Newbould305_1662	Staphylococcal secretory antigen Ssa2	Immunogenic protein of unknown function	Lang et al., 2000; Dubrac and Msadek, 2004
Newbould305_1498	Putative transcriptional regulator LytR	Cell wall organization	Sharma-Kuinkel et al., 2009
Newbould305_1676	Putative transcriptional regulator LytR	Cell wall organization	
**CELL WALL, MEMBRANE AND ENVELOPE BIOGENESIS**			
**Resistance**			
Newbould305_2227	Penicillin-binding protein 2 (PBP2)	Response to antibiotics	Lowy, 2003
Newbould305_0327	Penicillin-binding protein 3 (PBP3)	Response to antibiotics	
Newbould305_1169	Penicillin binding protein 4 (PBP4)	Response to antibiotics	
Newbould305_1499	Protein FmtA	Affects the methicillin resistance level and autolysis	Komatsuzawa et al., 1999
Newbould305_1724	Membrane-associated protein TcaA	Response to antibiotics	Maki et al., 2004
**Envelope biogenesis**			
Newbould305_0797	Teichoic acid biosynthesis protein F	Cell wall organization; Teichoic acid biosynthetic process	Fitzgerald and Foster, 2000
Newbould305_1248	Lipoteichoic acid synthase (LTA synthase)	Catalyzes the polymerization of lipoteichoic acid (LTA) polyglycerol phosphate	Karatsa-Dodgson et al., 2010
**INFORMATION STORAGE AND PROCESSING**			
Newbould305_1067	DNA-directed RNA polymerase subunit beta	DNA-directed 5′-3′ RNA polymerase activity	Wichelhaus et al., 1999
**METABOLISM**			
Newbould305_1866	Serine/threonine-protein kinase PrkC	Cellular response to peptidoglycan; Spore germination	Debarbouille et al., 2009
**MOONLIGHTING PROTEINS**			
Newbould305_0110	Peptidoglycan endo-beta-N-acetylglucosaminidase	Hydrolase activity	Heilmann et al., 2003, 2005
Newbould305_1307	Glyceraldehyde-3-phosphate dehydrogenase	Glycolysis	Modun and Williams, 1999
Newbould305_1311	Enolase	Catalyzes the reversible conversion of 2-phosphoglycerate into phosphoenolpyruvate	Antikainen et al., 2007
Newbould305_1073	Elongation factor Tu (EF-Tu)	GTPase activity	Widjaja et al., 2017

### *S. aureus* N305-secreted EVs are not cytotoxic against bMEC *in vitro*

**Figure 3:**
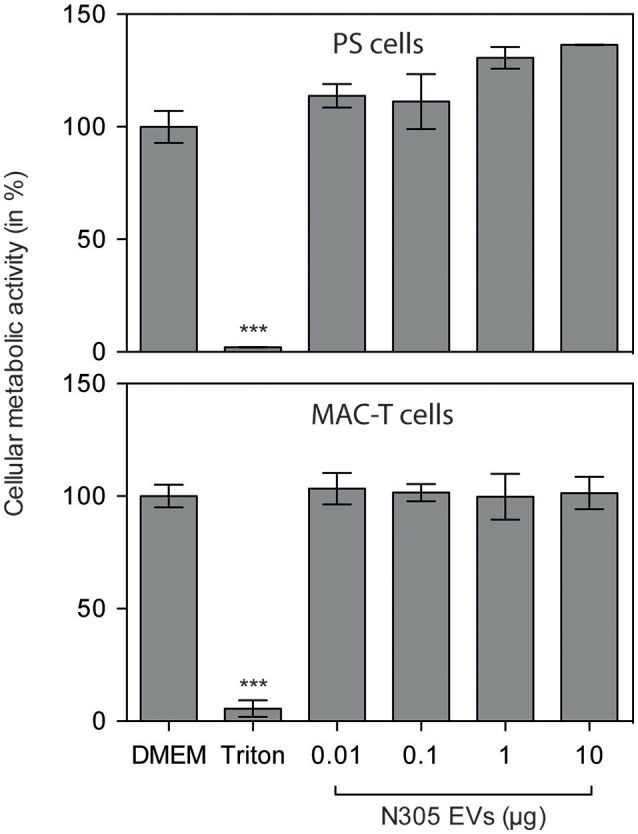
*S. aureus* N305-secreted EVs are not cytotoxic *in vitro* on MAC-T and PS bovine mammary epithelial cells. Either MAC-T or PS cells were treated with different EVs doses: 0.01, 0.1, 1, and 10 μg for 24 h. DMEM alone was used as mock control. Cellular metabolic activity was evaluated by MTT. The results are shown as the percentage of the control. Data are presented as mean ± SD. Each experiment was done in triplicate. The differences among the groups were assessed by ANOVA. Tukey's Honestly Significant Difference test was applied for comparison of means. No cytotoxic effect of EVs in MAC-T or PS cells was observed after 24 h of treatment. ***P < 0.0005.

We first evaluated whether *S. aureus* N305 EVs could affect the viability of eukaryotic cells. For that purpose, two bovine mammary epithelial cell (bMEC) lines, MAC-T and PS were treated for 24 h with growing EVs doses: 0.01, 0.1, 1 and 10 μg per well. The analysis of viability by MTT analysis did not reveal any differences between the MAC-T and PS control cells and the cells exposed to EVs (Figure 3). These results show that *S. aureus* N305 EVs do not induce the cytotoxic effect in both MAC-T and PS cells in the tested conditions (Figure 3).

### *S. aureus* N305-secreted EVs induce an immunostimulatory response in bMEC *in vitro*

**Figure 4:**
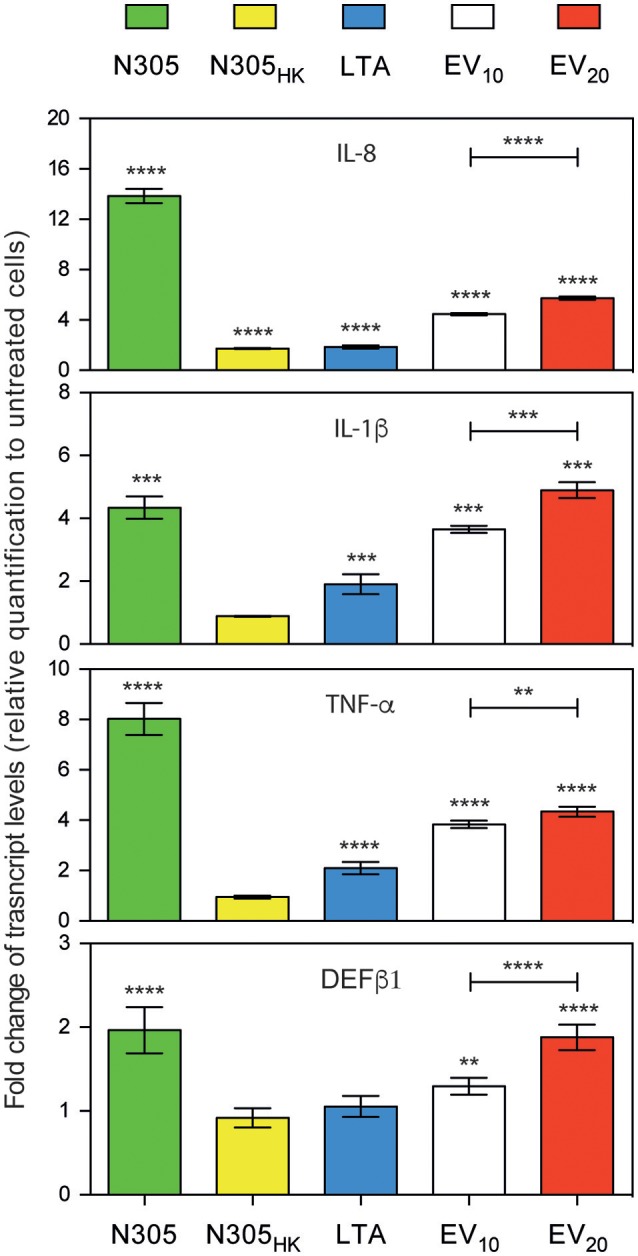
*S. aureus* N305-secreted EVs induce an immunostimulatory response *in vitro* on PS bovine mammary epithelial cells. Expression of IL-1β, IL-8, TNF-α, and DEFβ1 by bovine mammary epithelial PS cells shown as fold changes at mRNA level measured by RT-qPCR after 3 h post stimulation with either viable *S. aureus* N305 cells (N305, green), heat-killed *S. aureus* N305 cells (N305_HK_, yellow), 10 μg of purified staphylococcal lipoteichoic acid (LTA, blue), 10 μg and 20 μg of N305 EVs (EV_10_, white; EV_20_, red). Values were calculated as the mean ± SD obtained from three independent experiments after normalization to mock control DMEM. Asterisks indicate statistical significance as evaluated by one-way analysis of variance (ANOVA). ****P < 0.0001; ***P < 0.0005; **P < 0.005.

To examine whether *S. aureus* N305**-**secreted EVs could induce the host's immunity *in vitro*, particularly the innate defense, the PS cell line was then treated with *S. aureus* N305-secreted EVs (10 and 20 μg per well). Live and heat-killed *S. aureus* N305 (MOI 100:1; 25 μg) and LTA, a pro-inflammatory component of the *S. aureus* envelope (von Aulock et al., 2003) were used as complementary positive controls. The expression levels of host genes coding for key pro-inflammatory cytokines (IL-1β, IL-8 and TNF-α) and for the antimicrobial peptides β defensing-1 (DEFβ1) were compared to those of untreated PS cells (Figure 4). A significant induction of all genes was observed after treatment with live *S. aureus* compared to untreated PS cells. In contrast, no differences in IL-1β, TNF-α, and DEFβ1 expression were observed following treatment with heat-killed bacteria, while the IL-8 expression was slightly (fold-change = 1.7) but significantly increased compared to untreated PS cells. Treatment with the positive control LTA increased the expression level of tested genes. Finally, we observed a significant and dose dependent increase of IL-8, IL-1β, TNF-α, and DEFβ1 expression level in presence of *S. aureus* N305 EVs (Figure 3, EV_10_ and EV_20_), either to a similar (IL-1β, DEFβ1) or slightly lower (IL-8, TNF-α) level than those following treatment of PS cells with live *S. aureus* N305. These results demonstrated the ability of *S. aureus* N305-secreted EVs to stimulate bMEC *in vitro* in a way similar to that of live bacterial cells.

### *S. aureus* N305-secreted EVs induce inflammatory and a local host innate immune response *in vivo*

**Figure 5:**
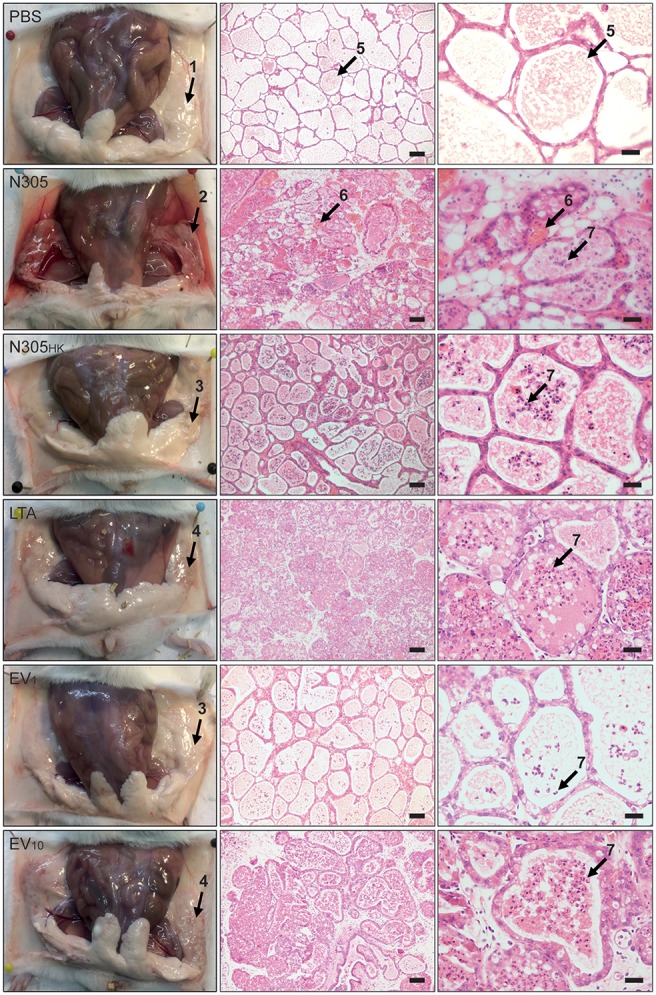
Histological consequences of inoculation of *S. aureus* N305-secreted EVs in murine mammary glands. Left panel: Gross pathology of mammary glands. Representative photographs from dissected mice are shown. Conditions are PBS treatment (PBS) (negative control group), viable *S. aureus* N305 cells (N305) (positive control group), heat-killed *S. aureus* N305 cells (N305_HK_) (positive control group), 10 μg of purified staphylococcal lipoteichoic acid (LTA) (positive control group), 1 μg of EVs (EV_1_) (test group) and 10 μg of EVs (EV_10_) (test group). Arrows emphasize different morphological and histopathological manifestations in the mammary gland post-treatment: 1, healthy lactating mammary gland; 2, severely inflamed mammary gland with bacterial exudates; 3, slightly inflamed mammary gland; 4, moderately inflamed mammary gland. Macroscopic differences resulting from the different treatments of the mammary glands are clearly visible (e.g., prominent redness and inflammation in the *S. aureus* N305, LTA and EV_10_ groups). Middle and right panels: Representative H&E stained tissue sections of the mammary gland from each group acquired at two magnifications are shown; middle panel: 20x, scale bar = 50 μm; left panel: 40x, scale bar = 20 μm. Arrow labeled 5 highlights the milk secrete in the alveolar lumen; arrows labeled 6 marks red blood cells; arrows labeled 7 marks immune cells in the lumen of alveoli. At 24 h p.i. the PBS group did not show any immune cell influx in the alveolar space, the *S. aureus* N305 group alveolar lumen had a profound hemorrhage and a stronger immune cell influx compared to the *S. aureus* N305_HK_ and LTA groups. The EV_1_ and EV_10_ groups had a dose-dependent recruitment of immune cells with an influx for EV_10_ similar to that observed in the LTA group.

To evaluate the *in vivo* modulation of *S. aureus* N305-secreted EVs on mammary gland inflammation, a well-defined experimental model of bovine *S. aureus*-induced mouse mastitis was used (Peton et al., 2016). Six groups of mice were inoculated with either PBS (negative control), live *S. aureus* N305 (first positive control), heat-killed *S. aureus* N305 (*S. aureus* N305_HK_, second positive control), LTA (third positive control), *S. aureus* N305-secreted EVs (1 μg, EV_1_ or 10 μg, EV_10_). At 24 h p.i., macroscopic signs of inflammation were observed in the glands that received live *S. aureus* N305, LTA and EV_10_ and in a much lesser extent in the glands that received *S. aureus* N305_HK_ and EV_1_. The mammary gland inoculated with *S. aureus* N305 had an average bacterial load of 8.94 ± 0.25 × log10 (CFU/g) at 24 h p.i. and showed a profound edema and hemorrhage. This severe clinical response was attenuated in the 2 other positive control groups (*S. aureus* N305_HK_ and LTA), and also in the EV_1_ and EV_10_ (Figure 5). Upon microscopical evaluation, a comparable influx of immune cells was observed in the alveoli of all treated mammary glands except for the PBS-inoculation (Figure 5). Of note, mammary glands treated with EV_1_ had less immune cells in their alveoli compared to EV_10_-inoculated mice again indicating a stronger inflammatory response for the higher dose.

**Figure 6:**
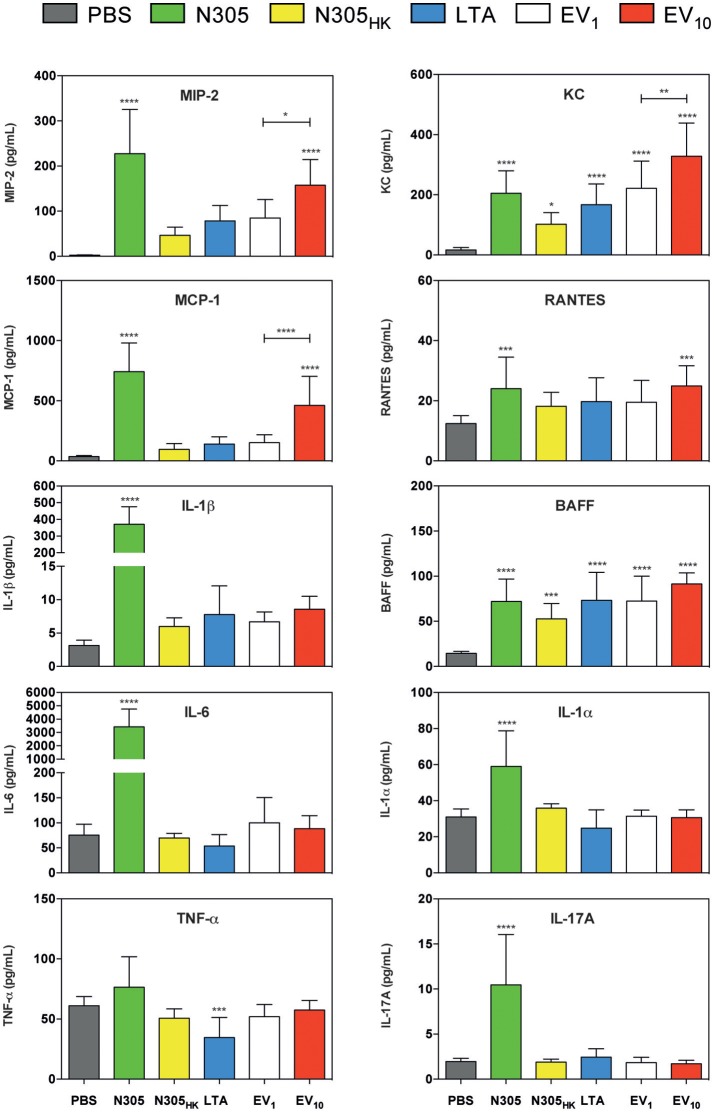
Immunological consequences of inoculation of *S. aureus* N305-secreted EVs in murine mammary glands. Cytokines were quantified from mammary gland lysates using multiplex immunoassay. Conditions are PBS treatment (PBS, gray) (negative control group), live *S. aureus* N305 (N305, green) (positive control group), heat-killed *S. aureus* N305 (N305_HK_, yellow) (positive control group), purified staphylococcal lipoteichoic acid (LTA, blue) (positive control group), 1 μg of *S. aureus* N305-secreted EVs (EV_1_, white) and 10 μg of EVs (EV_10_, red). EVs induced significantly the secretion of MIP-2, MCP-1, KC, RANTES, and BAFF. The induction of MIP-2, KC and MCP-1 secretion was dose-dependent. The secretion of the cytokines IL-1α, IL-1β, IL-6, and IL-17A was only induced by *S. aureus* N305. The secretion of TNF-α was only induced by LTA. Asterisks indicate statistical significance compared to the negative control (PBS) as evaluated by one-way analysis of variance (ANOVA). ****P < 0.0001; ***P < 0.0005; **P < 0.005; *P < 0.05.

The local levels of cytokines IL-1α, IL-1β, IL-6, MCP-1 (CCL2), IL-17A, RANTES (CCL5), BAAF, MIP-2, and KC (CXCL1) were significantly higher in the mammary glands inoculated with live *S. aureus* N305 compared to PBS (Figure 6). Inoculation with *S. aureus* N305_HK_ and LTA also induced some of these cytokines (i.e., BAFF and KC) but this increase was much more modest (LTA: BAFF and KC *P* < 0.0005; *S. aureus* N305_HK_: BAFF *P* < 0.0005 and KC *P* < 0.05). *S. aureus* N305-secreted EVs significantly induced several local cytokine levels i.e., MCP-1 (CCL2), RANTES (CCL5), KC, MIP-2 and BAFF compared to PBS. In addition, the increase of MCP-1, BAFF, MIP-2, and KC appeared to be dose-dependent. The local IL-1β level showed a modest and also a dose-dependent increase, albeit non-significant compared to the PBS control. In terms of chemokines, *S. aureus* N305-secreted EVs elicited even a stronger local response than both the *S. aureus* N305_HK_ and LTA positive controls: for EV_10_ the average BAFF level was only slightly lower to that in live *S. aureus* N305-injected glands (91 ± 12 vs. 72 ± 25 pg/mL), while average KC levels (328 ± 110 vs. 205 ± 75 pg/mL) were even higher. These *in vivo* results demonstrated that *S. aureus* N305-secreted EVs induce a predominantly chemotactic local immunostimulatory response.

## Discussion

The Gram-positive pathogen *S. aureus* infects a wide range of tissues and is one of the most important bacteria in bovine mastitis negatively affecting milk production worldwide (Peton and Le Loir, 2014). The different degrees of clinical manifestations can be correlated to inter-strains variations in terms of specific virulence factors (Le Maréchal et al., 2011b). Furthermore, our knowledge of the host-pathogen interactions, as well as the molecular basis associated with persistence of *S. aureus* infections remains to be fully elucidated. Although EVs have been associated with multiple *S. aureus* infectious processes this is not yet the case in veterinary medicine (Hong et al., 2011; Kim et al., 2012). Thus, our objective was to investigate if EVs are secreted by the bovine udder isolate *S. aureus* N305 and their role in the context of mastitis.

*S. aureus* N305 secreted EVs that displayed the basic features of extracellular prokaryotic membrane vesicles, i.e., a nanometric size range and a cup-shaped morphology and spherical structure (Raposo and Stoorvogel, 2013). In terms of their protein cargo, they appeared to be enriched with lipoprotein and membrane protein classes as also shown for human *S. aureus* strains and other bacterial species (Deatherage and Cookson, 2012; Rath et al., 2013; Brown et al., 2014; Askarian et al., 2018). Furthermore, *S. aureus* N305-secreted EVs share several proteins in common with those of *S. aureus* strains isolated from human clinical sources (Lee et al., 2009; Jeon et al., 2016) (Supplementary data, Figure S1 and Table S3), supporting the hypothesis that conserved regulatory mechanisms for cargo sorting may exist. A remarkable feature of *S. aureus* N305-secreted EVs was the predominance of virulence factors that accounted for approximately 10% of their vesicular proteome. Similar observations exist for other pathogenic Gram-positive bacteria, such as *Mycobacterium tuberculosis* (Lee et al., 2015), *Bacillus anthracis* (Rivera et al., 2010), *Streptococcus pneumoniae* (Olaya-Abril et al., 2014), *Listeria monocytogenes* (Lee et al., 2013a), and *Clostridium perfringens* (Jiang et al., 2014). This feature suggests that virulent protein delivery via EVs represents an important common mechanism in the development or progression of infections. Consistent with this, in the current study proteins involved in key steps of mastitis pathogenesis such as adherence to host tissues, development of lesions and tissue damage, and evasion from the host immune system were observed. *S. aureus* N305-secreted EVs also contained numerous proteins associated with metal ion acquisition, a mechanism essential for local bacterial proliferation and for circumventing nutritional immunity, as well as proteins involved in resistance to antimicrobial agents. Additionally, several moonlighting proteins with secondary roles closely related with pathogenesis (e.g., enolase, GAPDH, autolysin, Tuf) (Modun and Williams, 1999; Heilmann et al., 2005; Antikainen et al., 2007; Widjaja et al., 2017) and lipoproteins involved in *S. aureus* Toll-like receptor 2 (TLR-2) activation and pathogenicity (Shahmirzadi et al., 2016) were identified. Collectively, our proteomic data suggest a role of *S. aureus* N305-derived EVs in the host-pathogen interaction.

Bovine mammary epithelial cells (bMECs) play an important role as the first line of defense against intramammary infections through the recognition of pathogens and the secretion of chemokines, cytokines and antimicrobial peptides that lead to neutrophil recruitment (Gray et al., 2005; Rainard and Riollet, 2006). Our *in vitro* data showed that bMECs exposure to *S. aureus* N305-secreted EVs led to a significant and dose-dependent increased expression of two pro-inflammatory cytokines (IL-1β, TNF-α), one chemokine (IL-8) and one bactericidal peptide (DEFβ1). Both cytokines are key elements of the early innate immune response in the mastitic mammary gland, comprising also the chemokine which is responsible for neutrophil recruitment and activation (Lahouassa et al., 2007), and the bactericidal peptide which is involved at the level of the oxygen-independent antimicrobial processes (Gurao et al., 2017). The ability to modulate the epithelial immune response has been described for EVs originated from both Gram-negative and -positive bacteria, including human *S. aureus* strains (Ismail et al., 2003; Bauman and Kuehn, 2006; Bomberger et al., 2009; Ellis and Kuehn, 2010; Kaparakis et al., 2010; Parker et al., 2010; Jun et al., 2017). Although the current study did not aim to unravel the molecular mechanism behind the response elicited by the *S. aureus* N305-secreted EVs, it observed no cytotoxicity on bMECs after 24 h of incubation. At first sight, this finding may seem unexpected given the abundance of virulence factors within these EVs. However, although they all harbor an arsenal of virulence factors, cytotoxic activity is not shared by all *S. aureus*-secreted EVs (Gurung et al., 2011; Thay et al., 2013; Jeon et al., 2016; Jun et al., 2017). The presence or absence of cytotoxicity may result from proteome differences between *S. aureus*-secreted EVs (Jeon et al., 2016). EVs produced by *M. tuberculosis* induce a TLR2-dependent pro-inflammatory response via their lipoprotein cargo in interacting directly with the plasma membrane receptor that stimulates intracellular signaling cascades (Prados-Rosales et al., 2011). A plausible hypothesis states that *S. aureus* N305-secreted EVs stimulate bMECs in a similar way, since they are also enriched in lipoproteins that are TLR2 ligands (Shahmirzadi et al., 2016). However, a variety of other mechanisms of action may exist. For example, *Helicobacter pylori*-secreted EVs exhibit NF-κB-dependent pro-inflammatory activities via inflammasome-dependent signaling through the cytosolic NOD1 receptor after their fusion with the epithelial plasma membrane and delivery of their cargo into the cytosol (Kaparakis et al., 2010). Another example is the pore-forming toxin cytolysin A delivered by *Escherichia coli* outer membrane vesicles which induces an epithelial pro-inflammatory response via alteration of the cellular Ca^2+^ homeostasis (Uhlén et al., 2000; Söderblom et al., 2005). Interestingly, *S. aureus* N305-secreted EVs also harbors toxins (PSMs, leukocidin) that are able to trigger Ca^2+^-mediated host cell activation (Barrio et al., 2006; Forsman et al., 2012). Whether these EVs act extracellularly through ligand-receptor interactions, intracellularly after their internalization, or by inducing subtle perturbations such as on the cellular Ca^2+^ homeostasis to modulate the epithelial immune and inflammatory response remains to be investigated. It will be of high interest to examine more closely the role of lipoproteins and toxins in this modulation.

Consistent with our *in vitro* results, intramammary inoculations with *S. aureus* N305-secreted EVs elicited a local response with a dose-dependent immune cell recruitment and the induction of a pro-inflammatory cytokine profile. LTA is a major immunostimulatory of Gram-positive bacteria and can induce secretion of cytokines *in vivo* (Fournier and Philpott, 2005; Rainard and Riollet, 2006). Of relevance, these EVs were able to induce a higher *in vivo* response than LTA and *S. aureus* N305_HK_ both at the histological and cytokine levels, which suggests their role in *S. aureus* N305 pathogenesis as immunostimulatory factors. The influx of inflammatory cells at inflammation sites is generally associated with elevated levels of CXC chemokines (Zlotnik and Yoshie, 2000). Accordingly, we detected an induction of the murine IL-8-like chemokines KC (CXCL1) and MIP-2 (CXCL2) reportedly involved in neutrophilic recruitment at inflammation sites (Rollins, 1997; Leemans et al., 2003; De Filippo et al., 2008). In addition, the levels of MCP-1, a monocyte chemoattractant (Rollins, 1997), RANTES, a monocytes, T cells, basophils and eosinophils chemoattractant (Arango Duque and Descoteaux, 2014) and BAFF, the B-cell-activating factor increased. The immune response induced by EVs was comparable to that observed with live *S. aureus* N305 although attenuated and not restricted to chemokine induction. Notably, the induction of IL-17, a critical cytokine for immune response and clearance of the pathogens at epithelial surfaces (Marks and Craft, 2009), was detected only with live *S. aureus* N305. These results showed that the immune response induced by *S. aureus* N305-secreted EVs might not be associated with IL-17-dependent T cell signaling. In addition live *S. aureus* N305 induced an increase production of several pro-inflammatory cytokines (i.e., IL-1α, IL-1β, IL-6), as previously reported (Breyne et al., 2014; Peton et al., 2016). These EVs appeared to mainly induce a chemotaxis related migratory response *in vivo* when compared to the responses induced by live *S. aureus* N305. This raises the question of the role of EVs and the biological significance of their pro-chemotactic effects during the *S. aureus* infectious process. In the infected udder, colonization and invasion of the mammary gland by bacteria is followed by a recruitment of polymorphonuclear neutrophilic granulocytes, which are responsible for clinical symptoms and determine the course of infection. *S. aureus* cells are able to survive within a variety of host cells including professional phagocytes such as neutrophils (Voyich et al., 2005) and monocyte-derived macrophages (Kubica et al., 2008) that may serve as a vehicle for persistence and dissemination of the infection (Garzoni and Kelley, 2009). One could view the chemotactic activity of *S. aureus* N305 EVs as a strategy to recruit phagocytic cells to allow the internalization of the bacterium and therefore its survival. This hypothetic strategy may also explain the persistence observed in *S. aureus* N305 infections. Additional studies are needed to better understand the role exerted by EVs in *S. aureus* pathogenesis, particularly with regard to strain-dependent clinical manifestations of mastitis and their involvement in chronic infection.

In summary, our study demonstrated at first that EVs are produced by the mastitis strain *S. aureus* N305 and that they induce an immunostimulatory response, both in bMEC *in vitro* and in a preclinical model of bovine mastitis. Furthermore, it provides evidence that *S. aureus* N305-secreted EVs principally modulate the chemotaxis of innate immune cells. These findings provide both novel insights in *S. aureus* mastitis pathogenesis and innovative avenues to control mastitis in which EVs are proposed as potential candidates for the development of vaccines as these are currently lacking in the treatment of Gram-positive udder infection.

## Author contributions

NRT, YLL, and EG designed the experiments. NRT, CC, KB, AD, KD, and JJ performed the experiments. NRT, JJ, KB, DC, EM, NB, VA, and EG performed the analyses. NRT, YLL, and EG wrote the manuscript and all of the authors critically reviewed the manuscript and approved the final version.

### Conflict of interest statement

The authors declare that the research was conducted in the absence of any commercial or financial relationships that could be construed as a potential conflict of interest.
