# Supplementary material for: Staphylococcus aureus Extracellular Vesicles Elicit an Immunostimulatory Response in vivo on the Murine Mammary Gland
Source: Front Cell Infect Microbiol. 2018 Aug 22;8:277. doi: 10.3389/fcimb.2018.00277 (PMC6113362; doi:10.3389/fcimb.2018.00277)
Supplement: Supplementary file 4 [file Presentation_1.PDF]

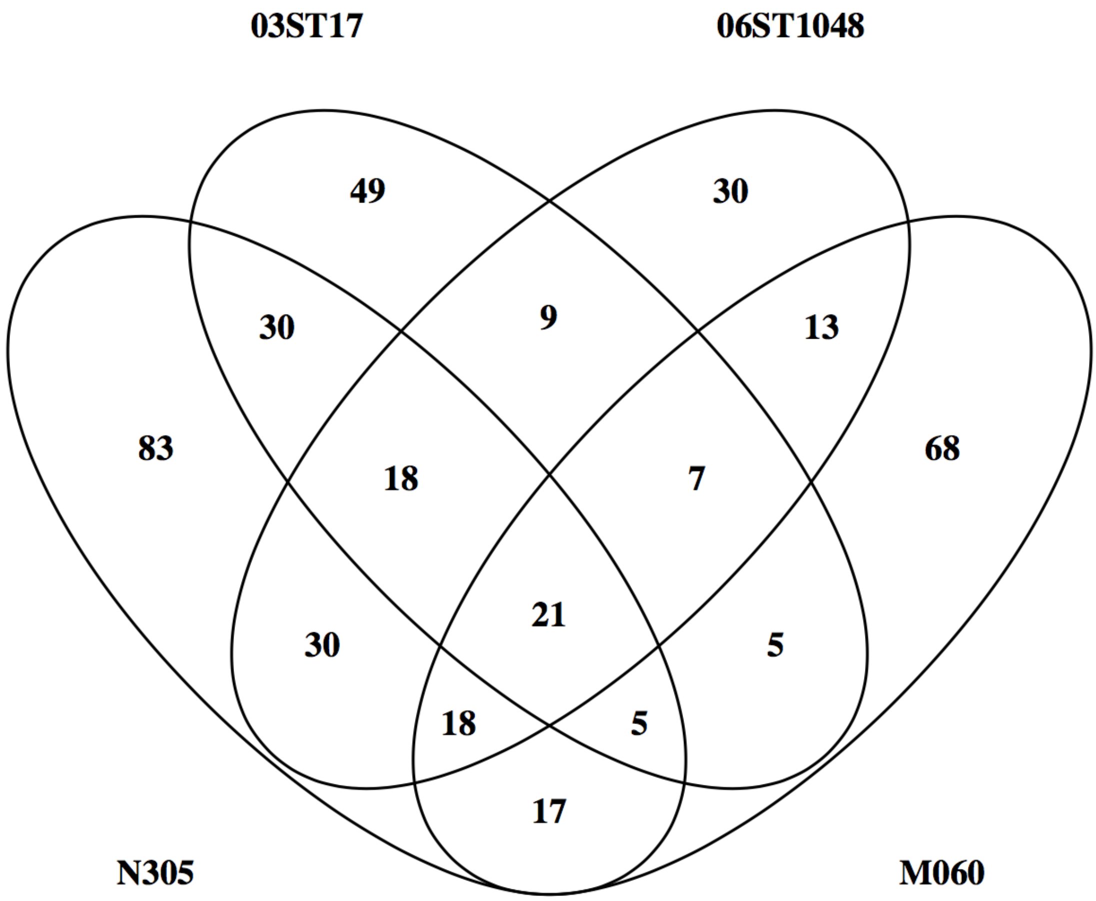

FIGURE S1. Venn diagram of proteins identified in EVs from *S. aureus* N305, 03ST17, 06ST1048 and M060 isolates. The number of proteins identified in the four different *S. aureus* EVs is presented. Proteins identified in EVs derived from *S. aureus* 03ST17, 06ST1048 and M060 were obtained from Jeon et al., 2016 (*Jeon H, Oh MH, Jun SH, Kim SI, Choi CW, Kwon HI, Na SH, Kim YJ, Nicholas A, Selasi GN, Lee JC. Variation among Staphylococcus aureus membrane vesicle proteomes affects cytotoxicity of host cells. Microb Pathog. 2016 Apr;93:185-93. doi: 10.1016/j.micpath.2016.02.014.*). Venn diagram was generated with Venny 2.1 (*Oliveros, J.C. (2007-2015) Venny. An interactive tool for comparing lists with Venn's diagrams*; http://bioinfogp.cnb.csic.es/tools/venny/index.html).
